# Wolves respond differently to human cues as they expand into urban landscapes

**DOI:** 10.1073/pnas.2529810123

**Published:** 2026-02-17

**Authors:** Martina Lazzaroni, Rudy Brogi, Francesca Brivio, Elena Bassi, Andrea Boromello, Tabea Teichmann, Friederike Range, Marco Apollonio, Sarah Marshall-Pescini

**Affiliations:** ^a^Domestication Lab, Konrad Lorenz Institute of Ethology, Department of Interdisciplinary Life Sciences, University of Veterinary Medicine Vienna, Vienna 1160, Austria; ^b^Department of Chemistry, Life Science and Environmental Sustainability, University of Parma, Parma 43124, Italy; ^c^Department of Veterinary Medicine, University of Sassari, Sassari 07100, Italy; ^d^National Biodiversity Future Center, Palermo 90133, Italy

**Keywords:** free-ranging wolves, neophobia, urbanization

## Abstract

Wolves (*Canis lupus*) recolonizing human-dominated landscapes encounter anthropogenic risks and opportunities, but how this shapes their fear of humans is unknown. We tested 185 individually identified wild wolves across 44 locations covering a wide urbanization gradient, exposing them to novel objects and human playbacks. Wolves from more urbanized areas showed reduced fear at first exposure to an object but heightened caution when the object changed, indicating increased attention to environmental changes, rather than uniform fear reduction. By contrast, playbacks of human voices elicited strong fear responses (>80%), regardless of urbanization. However, individuals habituated to both stimuli quickly and at a similar pace, highlighting their rapid learning abilities. Sociality buffered fear, with wolves in groups reacting less fearfully than wolves alone. Results show that wolves are capable of flexibly modulating their behavioral responses to both the risks and opportunities of human-dominated landscapes, which likely underpins their urban success and requires us to rise to the challenge of peaceful coexistence.

## Significance

With experimental tests on individually recognized wolves conducted in the wild, we provide unique insights into how wolves are adjusting their behavior in more heavily anthropized areas. Conducted in one of the regions where wolf colonization of human-dominated landscapes began earliest and has progressed furthest, we show that wolves in more urbanized areas become less wary of novelty but also more vigilant toward changes in their environment, while still retaining a strong, pervasive fear of cues indicating direct human presence (voices). These results highlight the complex, context-dependent nature of wolves’ fear and its variation along the urbanization gradient, offering a window into the future of wolf–human coexistence.

Humans have long perceived the wolf (*Canis lupus*) with ambivalence: a symbol of wilderness admired for its strength, intelligence, and hunting prowess, yet also a symbol of evil, feared and perceived as a harbinger of danger and chaos (1). This ambivalence may be reciprocated: for wolves, humans may be both a risk and a potential resource.

Human-caused mortality accounts for the majority of wolf deaths across Europe and North America (2, 3) and highly urbanized landscapes are particularly hazardous (4). Indeed, numerous studies have shown that wolves adjust their spatiotemporal activity patterns to minimize exposure to human activity (5, 6) and a recent experimental study demonstrated that wolves exhibit a significant fear of humans, who are regarded as a “superpredator” (7). Yet, in Europe, where wolves have recently recolonized much of their historical range and are entering densely urbanized landscapes (8–10), there is growing evidence that wolves can make good use of human-associated resources (11), for instance by shifting foraging habits toward hunting of domestic and pet animals (12, 13) and scavenging off human refuse (14), sometimes preferring these options to hunting their natural prey (15).

The exploitation of the anthropogenic landscape may prompt changes in animals’ fine-scale behavioral responses; however, the direction of such changes is not always consistent both within and across species. In few birds and mammal species, including widespread and long-time urbanized Canids like coyotes (*Canis latrans*) and red foxes (*Vulpes vulpes*), individuals in more urban environments show reduced neophobia when interacting with artificial objects (16–18) and less fearful responses when encountering humans (16, 18, 19). This may facilitate individuals colonizing human-dominated landscapes [henceforth “Colonization Hypothesis” (20)]. However, a few studies also show an opposite trend with individuals in more urban areas showing heightened caution toward novelty (21, 22) and maintaining high fear of humans (23), which could help protect them from potential threats and be especially advantageous for species facing high risks from humans [“Dangerous Niche Hypothesis” (24)]. A species’ sociality may further facilitate their presence in human landscapes, since it can mediate fearful responses by allowing individuals to rely on the supportive presence of partners when assessing novelty (25, 26) or potentially risky situations (27).

Given the long and intertwined history between wolves and humans, marked by domestication (28), enduring conflict leading to persecution (29), large-scale recovery (8), and the recent increased presence in densely human-populated landscapes (9), it is particularly timely to understand whether living in a more intensely human landscape is altering wolves’ behavioral responses.

**Fig. 1.**
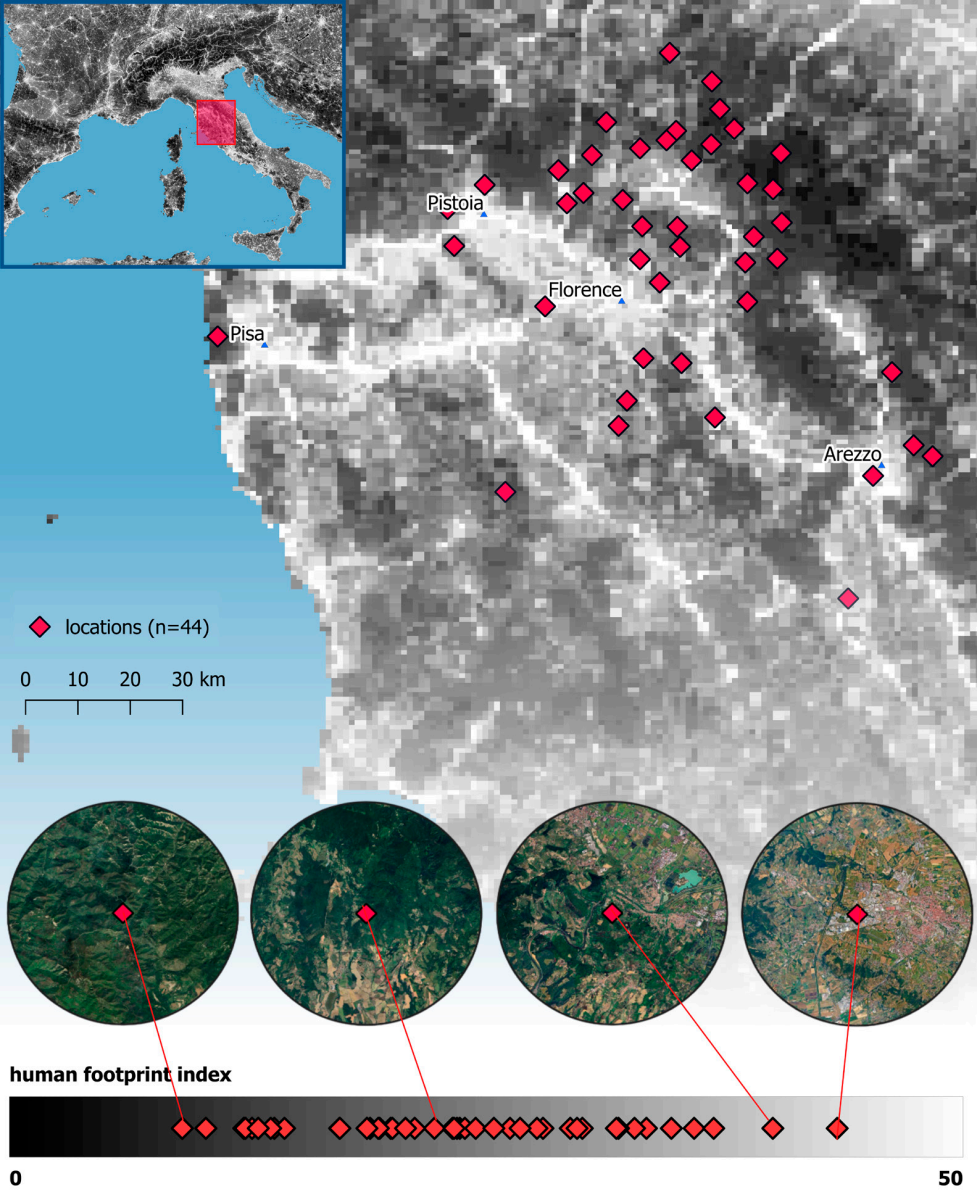
Distribution of test locations across central Italy. The *Upper-Left* panel shows a map of Italy, with a red rectangle indicating the study region. The main panel shows the spatial distribution of the 44 test locations (red diamonds) across central Italy along an urbanization gradient measured by the Human Footprint Index (HFI), ranging from 0 (black, lowest urbanization) to 50 (white, highest urbanization). At the *Bottom*, 4 circular panels provide representative examples of test locations with satellite imagery, illustrating the surrounding landscapes within the circular area used to calculate the local HFI.

**Fig. 2.**
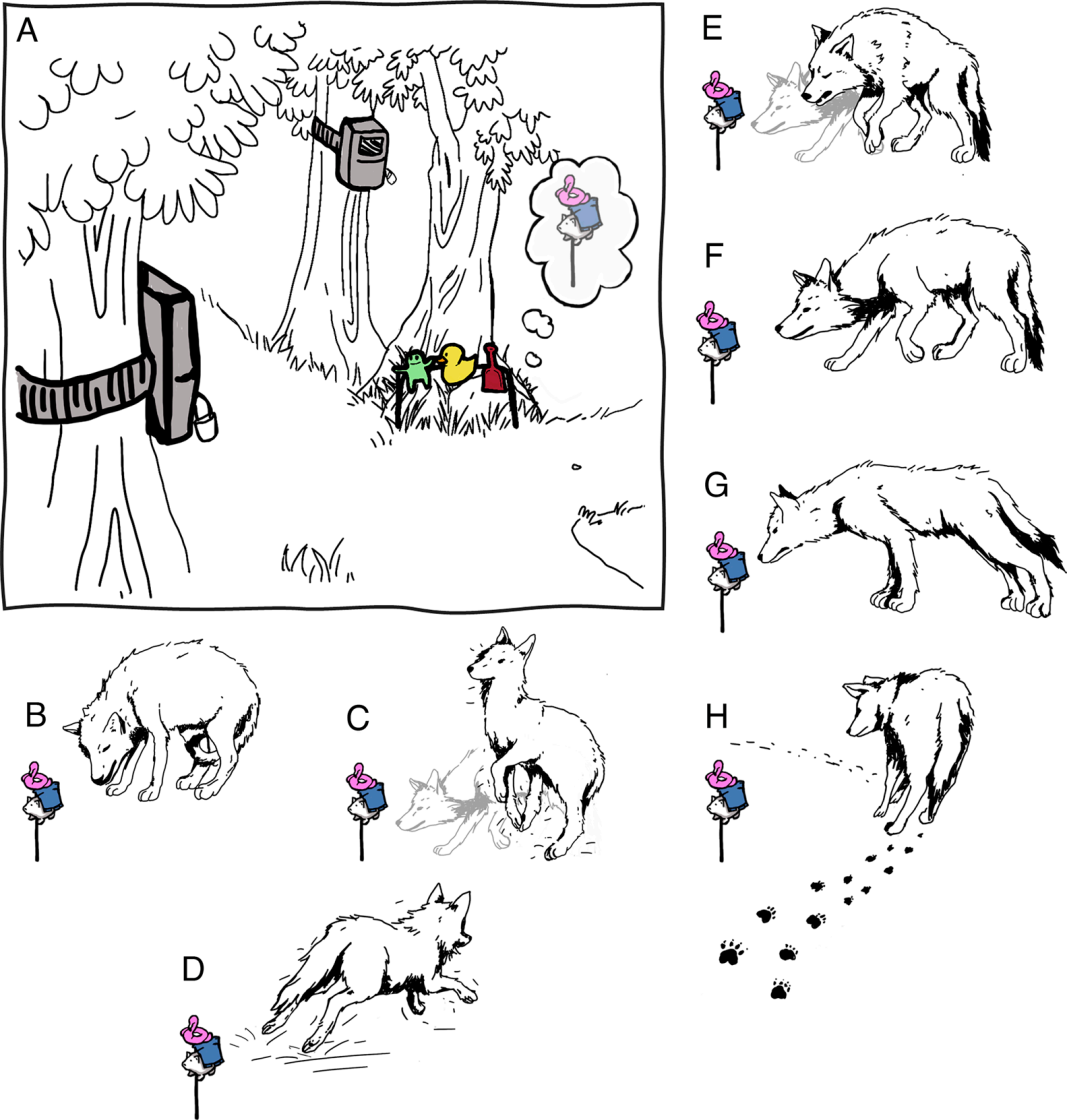
Experimental setup and behavioral metrics. (*A*) Graphic representation of the test setting. It shows an example of Object2 positioned on the ground and, in the thought bubble, an example of Object1. Object1 had been placed approximately 1 m from the portrayed position, a month earlier. (*B*) Insecure posture. (*C*) Startle. (*D*) Flee. (*E*) Wince. (*F*) Slow walk. (*G*) Risk assessment. (*H*) Avoidance. Fearful behaviors are portrayed in relation to the object but were similarly coded also in the playback test (see *SI Appendix*, Table S1 for detailed descriptions).

In this study, we tested a total of 185 individually recognized wild wolves with two tests assessing their response to both indirect and direct cues of human presence along the urbanization gradient. Wolves were tested in a total of 44 locations in Central Italy distributed along the urbanization gradient (Human Footprint Index; HFI 9.55 to 43.76, on a 0 to 50 scale) (Fig. 1). Two test sites (~800 m apart) per location were used, both fitted with two motion-sensitive video camera traps to allow for detailed behavior analyses. In the first test site, after a one-month camera-trap-only baseline, wolves were sequentially exposed to two slightly different novel objects for one month each (Object1 and Object2; Novel Object test: Fig. 2 and Movie S1). By allowing for repeated exposures to each object and presenting two different objects in succession, we assessed the animals’ speed of habituation to the stimuli and generalization/specificity response when exposed to a different, albeit similar object (30). On average two months later, in the second site, wolves were exposed to motion-triggered playbacks of human voices and bird vocalizations (the latter as control stimuli), each repeated twice and separated by two-week silent baseline periods (Movie S1).

Based on the wolves’ posture and marking behaviors (31), we categorized each individual as either being a breeder or not. Since wolf packs are largely composed of the breeding pair and their offspring, this allowed us to use this variable as a proxy to control for age, which can affect subject’s neophobia (32). We also controlled for potential effects of localized human activity by including the frequency of human passages at each site.

In line with other Canid urbanization studies (16, 17, 33), we predicted a lower likelihood of fear responses the higher the urbanization gradient in both tests. However, considering the high risk humans pose to wolves, we also predicted that they would a. generalize less from Object 1 to Object 2 the higher the urbanization gradient, thereby showing more vigilance to renewed novelty in the more urban landscapes, b. be more likely to respond with fear to human voices (signaling direct human presence) than to novel objects and in line with this, c. habituate faster to the presence of a novel object than to human voices. Finally, considering both the overall importance of sociality for wolves (34) and studies showing its buffering effect on the fear of novelty (25), we also predicted that wolves encountering the stimuli in a group would show a lower likelihood of fear than wolves encountering them alone.

## Results

### Urban Wolves Are Less Likely to Show Initial Fear Toward a Novel Object, But They Are More Cautious Than Wilder Wolves When a Slightly Different Object Is Presented.

**Fig. 3.**
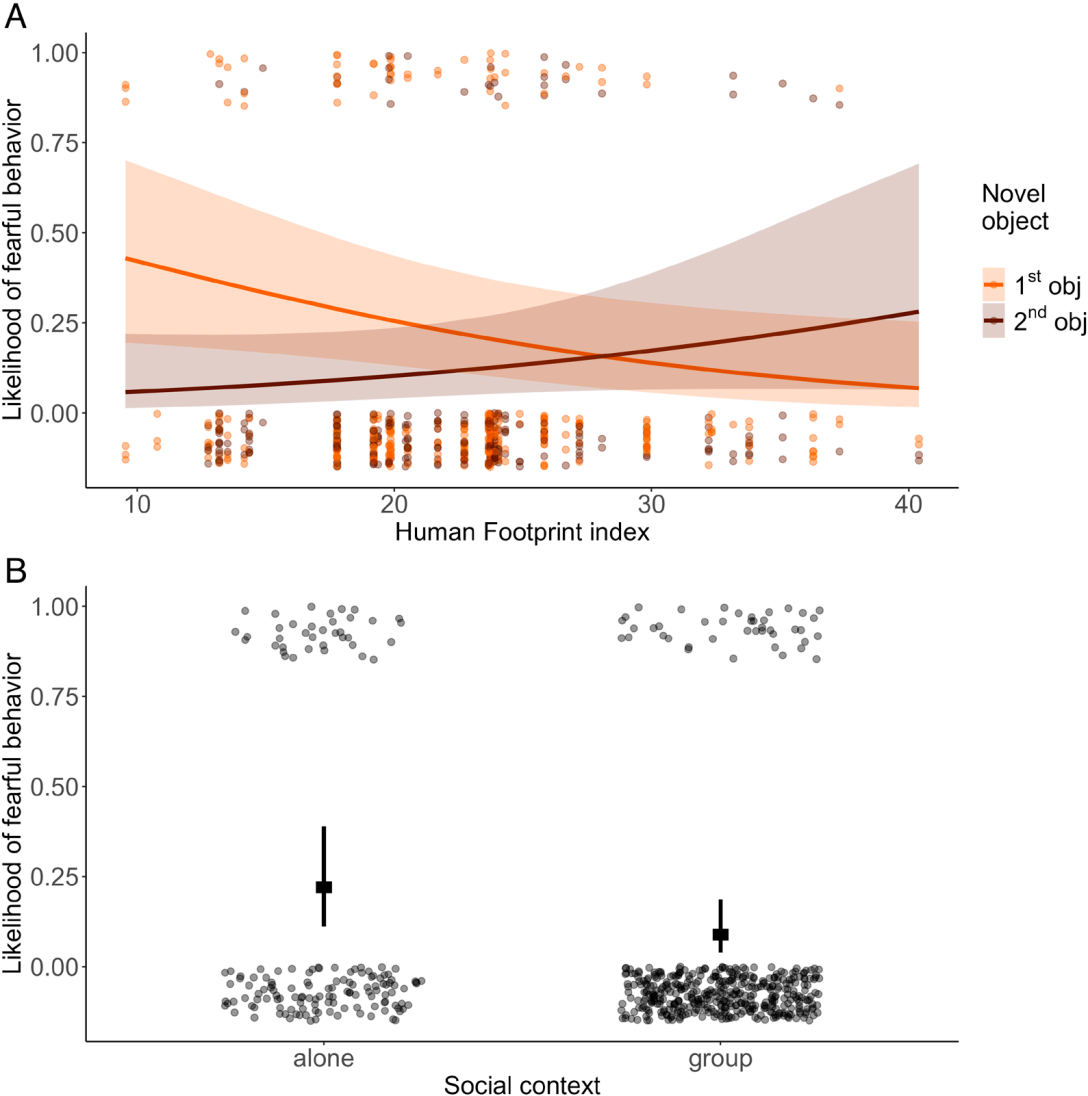
Fearful behaviors in response to novel objects. (*A*) Likelihood of occurrence of fearful behaviors along the urbanization gradient in the Novel Object test for Object1 (orange) and Object2 (brown). (*B*) Likelihood of occurrence of fearful behaviors in individuals encountering the objects alone or with at least one other social partner (group). In both panels, dots show observed values (with vertical jitter added to reduce overlap and ease the visualization), and lines indicate model estimates with 95% CI.

During the last two weeks of the camera-trap-only baseline period, fearful behaviors were extremely rare (3.1%) and were not affected by the urbanization gradient (β = 0.402 ± 0.394, z = 1.021, *P* = 0.307). When the 159 individually recognized wolves were exposed to the novel objects, the overall likelihood of a fearful response was quite low, occurring in 15% of all records for Object1 and 11% of all records for Object2 (N = 587 records each representing a single individual passage in front of the camera trap). Urbanization affected wolves’ likelihood of showing a fearful response (full-reduced model comparison: χ^2^= 6.155, df = 1, *P* = 0.013). When exposed to Object1, wolves were less likely to exhibit a fearful response the higher the urbanization gradient. However, interestingly, when exposed to Object2, in wilder areas wolves were less fearful of it than of Object1, but in more urbanized areas likelihood of showing fear toward the two objects remained the same (Fig. 3*A*). Habituation to the stimuli occurred within each condition (Object1 and Object2), with wolves showing a decreasing likelihood of fear as a function of exposure to the stimuli (“event number”: β = −0.926 ± 0.250, z = −3.705, *P* < 0.001). Regardless of test condition (Object1 or 2), wolves showed a higher likelihood of a fear response when they encountered the object alone than with a social partner (β = 1.159 ± 0.360, z = −3.222, *P* = 0.001; Fig. 3*B*). However, we found no effect of age/status on the likelihood of showing fear (β = −0.163± 0.363, z = −0.449, *P* = 0.654) (*SI Appendix*, Table S3).

### Wolves Fear Human Voices More Than Bird Vocalizations, Regardless of Urbanization.

**Fig. 4.**
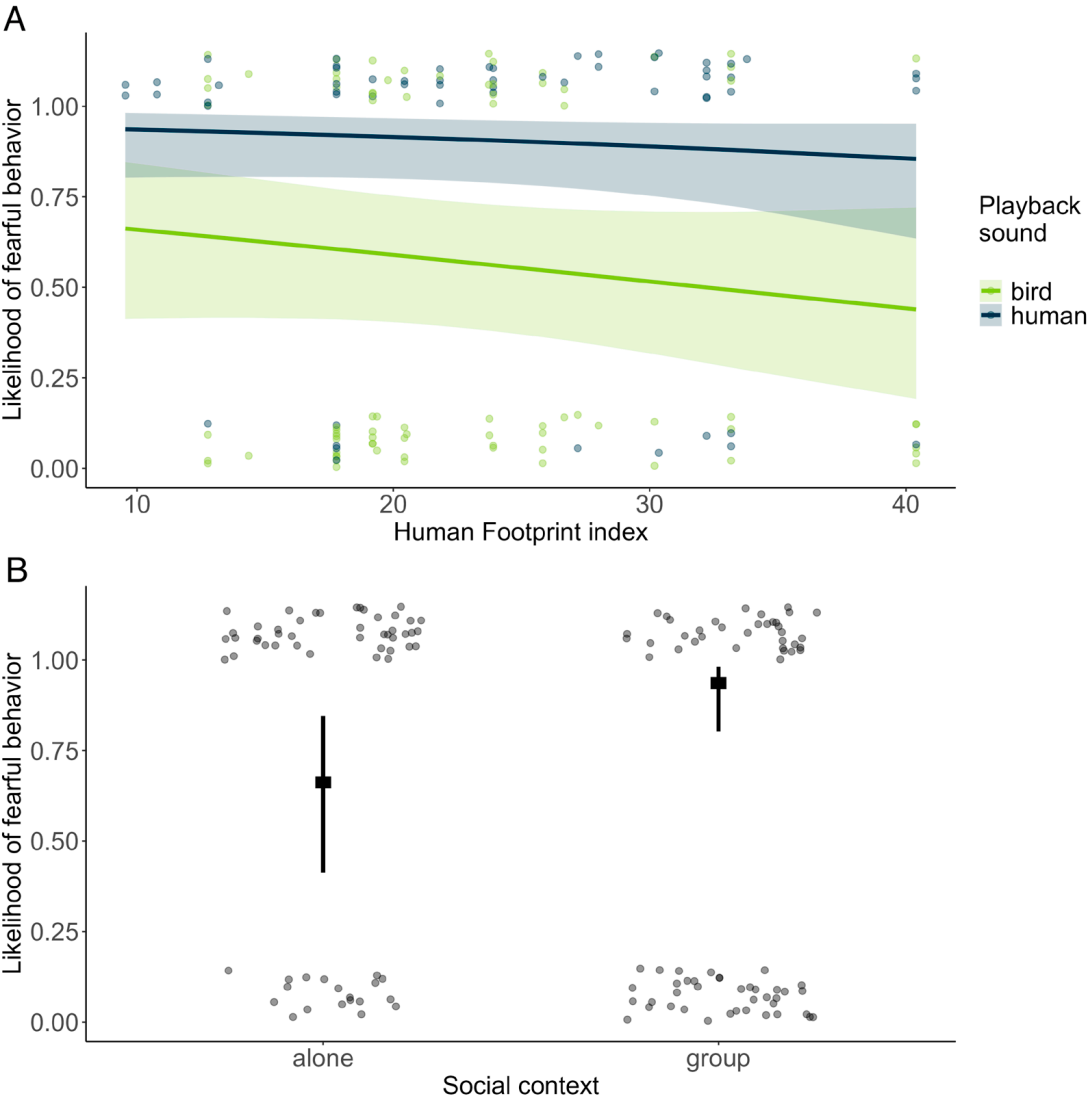
Fearful behaviors in response to acoustic stimuli. (*A*) Likelihood of occurrence of fearful behaviors along the urbanization gradient in response to the bird vocalizations (green) and human voices (dark blue). (*B*) Likelihood of occurrence of fearful behaviors in individuals encountering the playback alone or with at least one other social partner (group). In both panels, dots show observed values (with vertical jitter added to reduce overlap and ease the visualization), and lines indicate model estimates with 95% CI.

Based on 58 individually recognized wolves, we found that the likelihood of a fear response was affected by condition (Human *versus* Bird in the reduced model: β = 2.017 ± 0.480, z = 4.207, *P* < 0.001). Fearful responses occurred in 81% of records after human voices and 39% after bird vocalizations. However, the urbanization gradient had no effect on the animal’s response (no HFI*condition interaction, full-reduced model comparison: χ^2^= 0.016, df = 1, *P* = 0.899; no HFI main effect in the reduced model: β = −0.233 ± 0.214, z = −1.088, *P* = 0.276; Fig. 4*A*). Wolves showed a reduced likelihood of a fear response the more often they were exposed to the stimuli (“event number” in the reduced model: β = −1.044 ± 0.256, z = −4.073, *P* < 0.001). Despite the high incidence of fear responses, we still found a strong effect of sociality with a higher likelihood of wolves showing a fear response when encountering the sounds alone than with at least one other social partner (reduced model: β = 1.190 ± 0.450, z = −2.644, *P* = 0.008; Fig. 4*B*) (*SI Appendix*, Table S4).

### Wolves Fear Human Voices More Than Novel Objects, But Habituate to Them Just As Quickly.

**Fig. 5.**
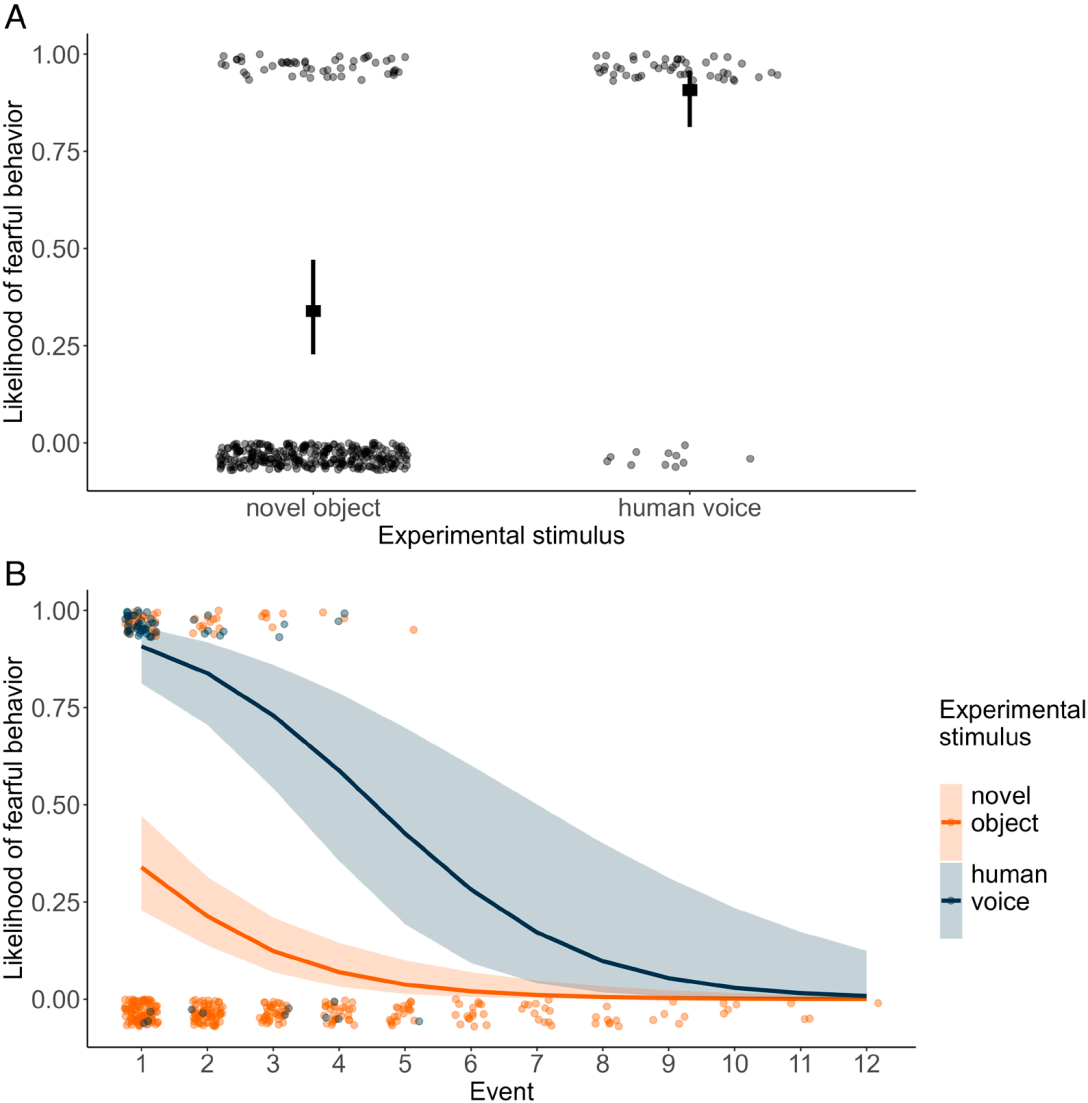
Fearful behaviors across tests: human voices versus novel objects. (*A*) Likelihood of occurrence of fearful behaviors in response to the first novel object (Object1 condition-Novel Object test, orange) and to human voices (Human voices condition-Playback test, dark blue). (*B*) Likelihood of occurrence of fearful behaviors with increasing numbers of exposures to the first novel object (orange) and human voices (dark blue). In both panels, dots show observed values (with vertical jitter added to reduce overlap and ease the visualization), and lines indicate model estimates with 95% CI.

To assess wolves’ fear of more direct and indirect cues of human presence, we compared the behavior of wolves when exposed to the first novel object (Object1) and to the human voices (Human voices) assessing whether the likelihood of exhibiting a fearful response and habituation patterns differed between the two tests. Overall wolves were more likely to exhibit fear when hearing the human voice compared to encountering the novel object (reduced model: β = 3.023 ± 0.419, z = 7.212, *P* < 0.001; Fig. 5*A*). In both tests wolves showed a reduced likelihood of exhibiting a fearful response over repeated exposures (reduced model: β = −1.503 ± 0.333, z = −4.520, *P* < 0.001; Fig. 5*B*), and, against our prediction, there was only a tendency for a different rate of habituation between tests (full-reduced model comparison: χ^2^ = 3.091, df = 1, *P* = 0.079; *SI Appendix*, Table S5), with a potentially faster habituation rate occurring with the novel object. The type of fear behaviors observed as a response to the two stimuli also differed. In the Novel Object test, the behavioral responses included in the majority of cases were exhibiting an insecure posture (lowered tail and hind quarters: 29% of cases) and/or avoidance/change of direction (deviating from the original path after looking at the object in order to increase the distance from self and object: 21%). Fleeing (running away) occurred in only 2% of cases. Human voices also frequently elicited an insecure posture (25%) and avoidance/change of direction (42%). However, running away, arguably a more extreme expression of fear, was observed in 35% of cases.

## Discussion

The vicinity of wild wolves to humans in such a densely populated world is a new reality. Our results show this vicinity has an effect on how wolves respond to differently “risky” human cues.

In the Novel Object test wolves displayed overall moderate levels of fear (medium: Object1 15% to Object2 11%) and quickly habituated to the presence of the objects, greatly reducing fearful responses after just a few passages. In line with the “Colonization Hypothesis” (and not the Dangerous Niche Hypothesis), wolves were less likely to show fear toward the first novel object in more urbanized areas. But when exposed to the second object, whereas wilder wolves showed a reduction in fearful responses, more urban wolves remained similarly cautious, providing some support also for the “Dangerous niche hypothesis”. Taken together, the differences in wolves’ responses to the two objects in urban vs. wilder environments portray a nuanced effect of urbanization. They suggest increased stimulus specificity (30) (i.e., a renewed response when a slightly different stimuli is presented) in more urbanized wolves, a population arguably exposed to higher risks, where increased attention to the details of the stimuli might be essential for subjects’ survival (35). We propose that rather than a blanket increase or decrease in overall fearfulness (as suggested by the Dangerous Niche and the Colonization hypotheses respectively), urbanization may increase the animals’ attention to changes in their environment, allowing individuals to evaluate and adjust their behavioral responses in relation to the perceived threat posed by each stimulus (“Urban Sensitivity Hypothesis”). Urban wolves strike a balance, with reduced neophobia facilitating exploration of the urban environment, while mitigating potential risks by heightened attention and caution toward environmental changes.

Wolves’ reaction to human voices, however, was consistently one of fear, regardless of the urbanization gradient. This aligns with previous finding showing wolves to significantly fear human voices more than control sounds and dog barks (7). Indeed wolves were much more likely to show a fear response to the human voices (>80%) than to the novel stimuli (15%), and reactions more often involved running away, considered indicative of imminent risk (36). Our findings align with previous experimental studies on urbanized animals, which report complex behavioral patterns characterized both by stimulus-specific responses (37, 38) and by multifaceted behaviors that reveal subtle differences in how responses are expressed (16). Together these studies highlight the nuanced responses animals employ to cope with more urban environments and suggest that behavioral adjustments are shaped by both population and context specific risk–benefit trade-offs. For wolves, considering the danger humans pose (3, 39), a generalized reduction in fearfulness could be maladaptive (40, 41), whereas the ability to modulate responses depending on the stimulus type may represent the key for successfully navigating the anthropogenic landscape (37).

In line with previous research on captive wolves (25), and with evidence from other species showing that social groups can mitigate fear and facilitate resource exploitation (42–44), our findings show that wolves encountering both stimuli with at least one other social partner were consistently less fearful than when alone. This indicates that social support can buffer neophobic responses in wolves, even under conditions of high immediate danger, such as human presence. We might speculate that alongside high dietary diversity (45), a comparably large brain (46) and advanced problem-solving skills (46), wolves’ pronounced sociality may further enhance their ability to thrive in human-shaped environments.

Wolves’ speed of habituation to both stimuli, including the human voice that initially elicited such a strong and consistent fear response, highlights their rapid learning abilities (47, 48). This may highlight why it is such a challenge to design effective deterrents for conflict management (49, 50). As contact between wolves and humans intensifies across the Western world (10), the need for systematic studies to understand how to increase the effectiveness of deterrents is badly needed to safeguard the fragile peacefulness of a relationship that is once again becoming central, yet still bears the weight of a long history of conflict.

Taken together, our results reveal wolves’ high potential to navigate urban environments as landscapes of both risks and opportunities, thanks to a multifaceted, flexible, and complex behavioral repertoire. The unresolved question is whether human societies can rise to the coexistence challenge with solutions of comparable effectiveness and complexity.

## Materials and Methods

Details of the study area and test setting, subjects, coding of tests, assessment of urbanization gradient and human disturbance, as well as statistical analyses carried out are included in the *SI Appendix*, *Materials and Methods* and Fig. S1 and Tables S1–S5 and Movies S1 and Datasets S1–S3. Language editing and refinement were assisted by ChatGPT (GPT-4.1; OpenAI), using prompts limited to requests for grammatical correction and improved clarity. All content was critically reviewed and approved by the authors. The project was reviewed by the Ethical Committee for Animal Experimentation of the University of Parma and was approved as exempt from regulated procedures under the Italian Legislative Decree 26/2014 (Protocol No. 03/CESA/2025).

## Supplementary Material

Appendix 01 (PDF)

Dataset S01 (XLSX)

## Data Availability

Movie S1; R scripts; R workspace data have been deposited in Figshare (https://figshare.com/s/a439bb50a8e50f1fb84b?file=55758653; https://figshare.com/s/a60d6fcbf09b0c882dd5;) (51, 52). Study data are included in the article and/or supporting information.
