# Supplementary material for: Wolves respond differently to human cues as they expand into urban landscapes
Source: Proc Natl Acad Sci U S A. 2026 Feb 17;123(8):e2529810123. doi: 10.1073/pnas.2529810123 (PMC12933097; doi:10.1073/pnas.2529810123)
Supplement: Supplementary file 1 — Appendix 01 (PDF) [file pnas.2529810123.sapp.pdf]

Supporting Information for

Wolves respond differently to human cues as they expand into urban landscapes

Martina Lazzaroni[1,2*†], Rudy Brogi[3,4†], Francesca Brivio[1], Elena Bassi[3], Andrea Boromello[3], Tabea Teichmann[1], Friederike Range[1], Marco Apollonio[3,4], Sarah Marshall-Pescini[1*]

[1] Domestication Lab, Konrad Lorenz Institute of Ethology, Department of Interdisciplinary Life Sciences, University of Veterinary Medicine Vienna, Vienna 1160, Austria.
[2] Department of Chemistry, Life Science and Environmental Sustainability, University of Parma; Parma, 43124, Italy.
[3] Department of Veterinary Medicine, University of Sassari; Sassari, 07100, Italy.
[4] National Biodiversity Future Center (NBFC); Palermo, 90133, Italy.
* Corresponding authors. Email: martina.lazzaroni@unipr.it; sarah.marshall@vetmeduni.ac.at
† These authors contributed equally to this work

*Corresponding authors: Martina Lazzaroni, Sarah Marshall-Pescini.
Email: martina.lazzaroni@unipr.it, sarah.marshall@vetmeduni.ac.at

This PDF file includes:

SI Materials and Methods
Figure S1
Tables S1 to S5
Legends for Movie S1
Legends for Datasets S1 to S3
SI References

Other supporting materials for this manuscript include the following:

Movies S1
Datasets S1 to S3

SI Materials and Methods

Study area and test setting

Data collection took place in Central Italy, between January and August of 2021, 2022, 2023, and 2024. We identified a total of 44 locations encompassing a broad range of urbanization levels in the landscape (1) (Table S1). Each location was composed of two test sites (one for Novel Object test and one for Playback test), except for 2021 when only the Novel Object test was conducted at a single site (68 test sites in total). The two test sites within the same location were 500–800 m apart, to increase the likelihood of testing the same subjects, whereas test sites from different locations were separated by at least 3.8 km (with the exception of two sites 2.4 km apart but divided by a high-traffic road), to minimize the risk of testing the same subjects in different locations. The data described above refer only to the locations included in the analyses, as we excluded sites where, after the initial baseline period, the same subjects were identified in different locations (this occurred on three occasions), as well as sites where individual identification was not possible (see section *Wolves' identity assessment*). All test sites were along human-used trails with the specific location chosen based on signs of wolf presence (scats, prints and hind-leg scratch marks). At each test site, we installed two no-glow trail cameras (Browning Spec Ops Advantage BTC-8A) to minimize the risk of data loss due to potential malfunctions and to capture more comprehensive footage of wolves' behaviors in response to the test stimuli. To ensure full coverage of the area, cameras were oriented in opposite directions. They were mounted on trees using cables and secured with padlocks. Depending on the specific features of each area, cameras were placed at heights ranging from 1 to 3 meters (2) and at a distance of 4 to 10 meters from the test stimuli (Novel Object test). Each camera was equipped with a 128 GB SD card and powered by an external 12V, 5A battery, both of which were replaced approximately every two weeks. Cameras were configured to record 1-minute videos upon activation by the PIR sensor. However, during nighttime hours, the maximum video length was automatically limited to 20 seconds. Initially, both cameras were active 24 hours a day. However, after a two-week monitoring period, if frequent false triggers caused by sunlight were detected, we adjusted the setup: one camera (camera A) continued operating 24/7, while the other (camera B) was set to a reduced schedule, excluding 2 to 6 hours of peak sunlight based on site-specific canopy cover and sun exposure (e.g., from 10:00 AM to 12:00 PM or from 10:00 AM to 4:00 PM). In the Playback test, the 24-hour camera was always the one equipped with the speaker.

Wolves' identity assessment.

Having excluded locations where the same individuals were present (see section above), we proceeded to identify individuals within each test location. Two observers independently watched all videos to assign individual IDs to wolves. Identification was based on morphological traits, including fur patterns, body size, head and ear shape, distinctive features (e.g., floppy ears, scars, blind eyes, missing limbs), tail base shape and position, and gait (3). When the observer could reliably distinguish a subject, the subject was assigned a unique ID label. Conversely, individuals for which identification was not possible were labeled as 'NA'. Inter-observer reliability on subject identification was carried out between the two observers on all datasets, excluding records in which the subjects were not visible (presence of fog, damaged videos, etc.). The dataset also included videos of additional behavioral tests, not included in this publication, but performed in the same locations between January 2021 and August 2024. Thus, inter-observer reliability was run on a total of 2969 records of wolf passages (including 1110 records for the Novel Object test and 616 records for the Playback test) resulting from a total of 1893 events. As multiple individuals could be visible during a single event, the number of records exceeds the number of events.

We calculated the percentage of agreement between the two observers for all records in which the most experienced observer (ML) assigned an individual ID (and which were subsequently included in the analyses), totaling 2062 records. The resulting agreement was 82.64%. In addition, the breeding status was assigned to each recognized individual and included in the analyses as a proxy for age (factor age/status). Breeding status was determined based on marking behaviors (i.e., raised-leg urination, defecation, ground scratching), and agreement between observers was 100% for records where both had identified the same individual.

Behavioral tests

Novel Object test. The apparatuses consisted of plastic children's toys of varying shapes and colors. In both Object1 and Object2, the set of toys measured between 15–20 cm in length and 10 cm in width. In Object1, the toys were mounted vertically on a straight iron rod, whereas in Object2 the set of toys was arranged horizontally at a height of 15–20 cm on an inverted U-shaped iron rod (Fig. 2). No measures were taken to remove human scent from the objects, which were handled with bare hands during installation. Each test site featured a unique combination of toys for each object. To reduce the risk of theft or vandalism, a sign was attached to each object explaining the purpose of the study and providing an email address for inquiries (4).

The procedure lasted 3 months (January-March) and included a baseline and two test conditions (Object1 and Object2). After a one-month baseline period in which only camera traps were placed at the test site, we placed Object1 at each site for one month (Object1 condition). At the end of this period, it was removed and replaced with Object2, which remained in place for another month (Object2 condition). Objects were positioned along the trail within the field of view of both camera traps but partially concealed by surrounding vegetation. This placement was intended to prevent wolves from spotting the object from a distance and reacting before entering the cameras' visual range. Object2 was always placed ~1 meter from Object1's original position.

Playback test.

The apparatuses consisted of an Automated Behavioural Response (ABR) system, called 'BoomBox'(5). This system consists of a circuit board connected directly to the PIR motion sensor of the camera trap, which triggers audio playback through external speakers when the PIR sensor is activated. At each test site, one of the two camera traps was linked to a BoomBox unit. Each BoomBox was programmed using Arduino software (6) with audio clips corresponding to either the Human voices condition or the Bird vocalization condition. Two sets of playback sounds (set A and B) were prepared for both conditions, each comprising five five-second audio clips. For the Human condition each set consisted of clips of different single male voices speaking in a neutral tone of voice. For the Bird condition, set A comprised audio clips of *Strix aluco* vocalization, whereas set B comprised audio clips of *Luscinia megarhynchos* vocalizations, both species native to the study area. Given that wolves are largely nocturnal in the region, we selected one nocturnal species and one species that can be heard both at night and during the day.

All audio clips were sourced from publicly accessible television programs, available as open-access video recordings, and matched for temporal duration and normalized at an average volume of 80 dB.

Overall, the procedure lasted 3 months from May-June to July-August of 2022 2023, and 2024.

Audio playbacks were triggered randomly, starting two seconds after PIR sensor activation of the camera trap, and with a minimum 20-second interval between consecutive playbacks. This ensured that if an animal remained in front of the camera, the next sound would not play immediately. Each sound set lasted for two weeks, and, if no wolf passages were recorded in that period, the presentation was extended to two additional weeks. After each condition a two-week silent baseline period followed, in which only camera traps, with no playback sound, were present. The order of sound sets (A or B) was randomized across sites, and the initial condition (Human or Bird) was counterbalanced across locations (so that 48% of locations started with the Human condition and 52% with the Bird condition). Human and Bird conditions were always alternated (fig. S1). After the exposure to the first two sound sets, the boombox was moved to the tree where the second camera was mounted. Additionally, after the first and third exposure to any of the sound sets, the orientation of the speakers was modified. These slight changes were introduced to mimic a more realistic scenario whereby bird vocalizations and human voices did not come from the exact same place every time, yet maintaining the same test-site. We presented the two sets of sounds for each condition, with multiple audio clips in each, to increase the probability that if the same animal passed by, they would not hear the exact same audio-clip, making the scenario more realistic. We repeated each condition twice to increase the chances of testing all individuals at least once in both test conditions.

Behavioral coding. Videos of the same events recorded from both camera traps were mounted together with the software VegasPro (7) and uploaded in BORIS (8) for behavioral coding (Table S2). Videos were considered as part of the same event if occurring within a maximum of 2 minutes. Inter-observer reliability was assessed between two experimenters using Cohen's Kappa, each coding the same 160 records for the Novel Object test (insecure posture: κ=0.89, $p<0.001$; startle response: κ=1, $p<0.001$; wince: κ= 0.737, $p<0.001$; slow walk: κ= 0.65, $p<0.001$; flee: κ=1, $p<0.001$; risk assessment: κ=0.6, $p<0.001$; avoidance: κ=0.647, $p<0.001$) and the same 92 records for the Human Voices test (insecure posture: κ=1.000, $p<0.001$; startle response: κ=0.944, $p<0.001$; wince: κ=0.903, $p<0.001$; slow walk: κ=0.655, $p<0.001$; flee: κ=0.905, $p<0.001$; change direction: κ=0.897, $p<0.001$; stillness: κ=0.977, $p<0.001$).

<u>Assessment of urbanization gradient</u>
At each test site, we quantified local urbanization using the terrestrial Human Footprint (1-km resolution, 1). This index integrates multiple proxies of human presence and activity (i.e., population density, built infrastructure, cultivated lands, transportation networks, and nighttime lights) into a continuous score from 0 (lowest) to 50 (highest; Fig.1).
To characterize urbanization at a spatial scale relevant to wolves, we computed a distance-weighted mean Human Footprint within a circular buffer centered on each test site (radius = 5.129 km; area = 82.65 km²), matching the average home-range derived from wolves' pack density in central Italy (3). A simple areal mean would over-represent peripheral zones because annular area increases with radius. To mitigate this, we assigned each pixel included in the circular buffer a weight (*w*) that decayed linearly with its distance (*d*) from the test site: $w_i = 1 - d_i/r$, where *r* is the buffer radius. The site-level urbanization metric was then the weighted mean,

$$\overline{HFI} = \frac{\sum_i w_i H_i}{\sum_i w_i}$$

with H_i the Human Footprint value of pixel *i*. This scheme gives greater influence to areas more likely to have been used by the observed wolves while down-weighting increasingly uncertain, more distant areas. Since each location comprised up to two test sites, we defined location-level urbanization as the arithmetic mean of the corresponding site-level weighted means and assigned this value to all wolf subjects observed within that location.

<u>Assessment of human passage rate</u>
To assess the level of anthropogenic disturbance from recreational activities and motor vehicles at each test site, we used data from camera traps. For consistency and reliability, only footage from camera traps that operated continuously 24 hours a day were included in the analysis. In cases where both camera traps at a site were active 24/7, we randomly selected one to avoid double counting or bias. Human passages were initially classified into hikers, bikers, horseback riders, skiers, cars, motorcycles, quads, snowmobiles, and work-related vehicles. However, passages involving motor vehicles were excluded from analyses, since wildlife is generally less disturbed by vehicles than by humans on foot (9), and we reasoned that the exposure to human voices (more likely from humans on foot than in vehicles) would be the potentially more relevant factor affecting results in our study. Hence for analyses, hikers, bikers, horseback riders, and skiers were grouped under a single category, termed "human passage rate". The frequency of human passage was calculated for each site as the total number of individual detections recorded by the selected camera trap over the entire study period, divided by the number of effective camera trap days (i.e., the number of days the camera was fully operational).

<u>Analyses</u>
Analysis of wolves' fearful responses during the initial baseline period. In the study areas wolves may already be accustomed to the presence of camera traps, which are often used by hunters or wildlife managers, however, since this may differ across locations, before any tests were carried out, we conducted a four-week baseline period during which only camera traps were deployed at the test sites. To ascertain whether wolves had habituated to the presence of the camera traps we assessed whether in the last two weeks of the baseline, wolves differed in their likelihood of expressing fear behaviors across sites as a function of the varying anthropogenic landscape

(HFI). To this end we run a GLMM model (model FearBasel) where we included as response the binary variable Fearful, set to be 1 whenever a wolf performed at least one of the following: insecure posture, startle response, wince, slow walk, flee; 0 otherwise (Table S2). We included the HFI of the test site as a predictor and the site as a random intercept effect. These analyses were run on the whole dataset, including also not identified subjects, for a total of 328 records.

Analyses of wolves' fearful responses to novel objects along the urbanization gradient. Data for the Novel Object test were collected in a total of 44 sites. We collected a total of 667 videos, 343 for Object1 condition and 324 for Object2 condition, for a total of 1173 records, since the same video could include multiple subjects, hence multiple records. However, we included in the analysis only the records where we could identify the subjects. Thus, the final analyses were run based on data from a total of 40 sites, 361 videos, 587 records (334 Object1, 253 Object2), 50 different wolf packs, 159 identified subjects (of which we could identify 89 as breeders and 26 as non-breeders), 158 records of wolves tested alone and 429 tested in a group, 73 subjects tested in both conditions. Wolves were considered to be tested in a group if more than one individual was present during the same event.
We investigated whether the likelihood of wolves exhibiting a fearful response between conditions (Object1 and Object2) and across sites, as a function of the varying urbanization gradient (HFI). To this end we ran a GLMM model (model FearNovObj) where we included as the response the binary variable Fearful, set to be 1 whenever the subject performed at least one of the following: insecure posture, startle response, wince, slow walk, flee, risk assessment, avoidance; 0 otherwise (Table S2). We included as fixed effects the test condition (Object1 and Object2), the HFI, and their interaction, the factors alone/group and age/status. We additionally included as control variables the event number (progressive number indicating the times the subject passed in front of the specific stimulus) and the human passage rate. The subject ID was included as a random effect.

Analyses of wolves' fearful responses to human voices versus bird vocalizations along the urbanization gradient. Data for the Playback test were collected in a total of 33 test sites. We collected a total of 286 videos, 133 for the test condition (Human voices) and 153 for the control condition (Bird vocalizations), yielding a total of 391 records, as multiple subjects could appear in a single video. Playback failures occurred in 19% of cases (N = 73) due to malfunctions in the BoomBox system, where the audio was not triggered by the activation of the PIR sensor. In an additional 29 cases, subjects left the camera's field of view prior to the playback onset, preventing the capture of their behavioral responses. As with the novel object experiment, we constructed a dataset including only records where individuals could be identified. This final dataset comprised 139 records (59 test condition, 80 control condition), representing 58 individually identified wolves in a total of 28 test sites. Of these, 63 records involved wolves tested alone, while 76 involved wolves tested in groups. 16 individuals were tested in both test and control conditions.
We investigated whether the likelihood of wolves exhibiting fearful behavior after the sound differed between conditions (Human vs. Bird) and across sites as a function of the varying urbanization gradient (HFI). To this end we ran a GLMM model (model FearHumVoices) where we included as response the binary variable Fearful, set to be 1 whenever the subject performed at least one of the following: insecure posture, startle response, wince, slow walk, flee, change direction, stillness; 0 otherwise (Table S2). We included as fixed effects the test condition (Human and Bird), the HFI, and their interaction, and event number and alone/group. Subject ID was included as a random effect. Due to the relatively small sample size, we did not include human passage rate and the factor age/status to avoid model overfitting and overcomplexity.

Comparative analysis of wolves' fearfulness to novel objects and human voices. We compared the wolves' responses to Object1 and Human voices to assess whether the likelihood of exhibiting fear behaviors and habituation rates to the stimuli (decreased likelihood of a fear response over time/exposure) was test-dependent (model FearNovObj-Voices). This analysis used data from identified subjects collected during the Novel Object1 (N=122) and during the Human voices (N=36) and was run on a total of 392 records (334 Object1, 58 Human voices). We fitted a GLMM model with Fearful as the binary response variable. Fixed effects included test type

(Object1 vs. Human voices), event, their interaction, and HFI. We also included human passage rate and the factor alone/group as control variables. Subject ID was included as a random effect.

All the models were fitted in R (version 4.4.3 10) using the functions glmer of the package lme4 (11). Prior to fitting the models, we z-transformed all the continuous variables (HFI, the human passage rate, and the event number) to achieve an easier interpretable model (12) and ease model convergence. We used the function vif of the R package car (13) for screening the predictors for collinearity and multi-collinearity, but no issues arose (14). Additionally, to keep type I error rate at the nominal level of 5% (15, 16), for each model, we checked whether it was appropriate to include random slopes. We included the random slope of event number within subject ID in the models FearBasel, FearNovObj, while no random slopes were identifiable for other models. We did not include the correlations among random intercepts and slopes since not being identifiable. We also attempted to include random effects for event and location, but the models did not converge. After fitting the models, we inspected the distribution of the individual specific deviations from the common intercept and slopes (BLUPs). We determined model stability by dropping levels of the random effects one at a time and comparing the estimates derived from models fitted on the respective subsets with those obtained for the full data set, this revealed the models to be fairly stable (Tables S3 to S5). As an overall test of the effect of the fixed effects and to avoid “cryptic multiple testing” (17) each full model was compared with a null model lacking: condition, HFI and their interaction, alone/group and age/status for the model FearNovObj; condition, HFI, and their interactions, event and alone/group for the model FearHumVoices; test, event, and their interaction for the model FearNovObj-Voices. For the full-null model comparisons we utilized a likelihood ratio test (R function ‘anova’)(18). If the full-null model comparison resulted in significance, we tested the significance of the interaction by comparing the full model with a reduced model lacking the interactions but otherwise identical to the full model. If the interaction did not result in significance, we removed it from the model to inspect the main effect of the predictors.

Figures

Figure S1. Playback test procedure.

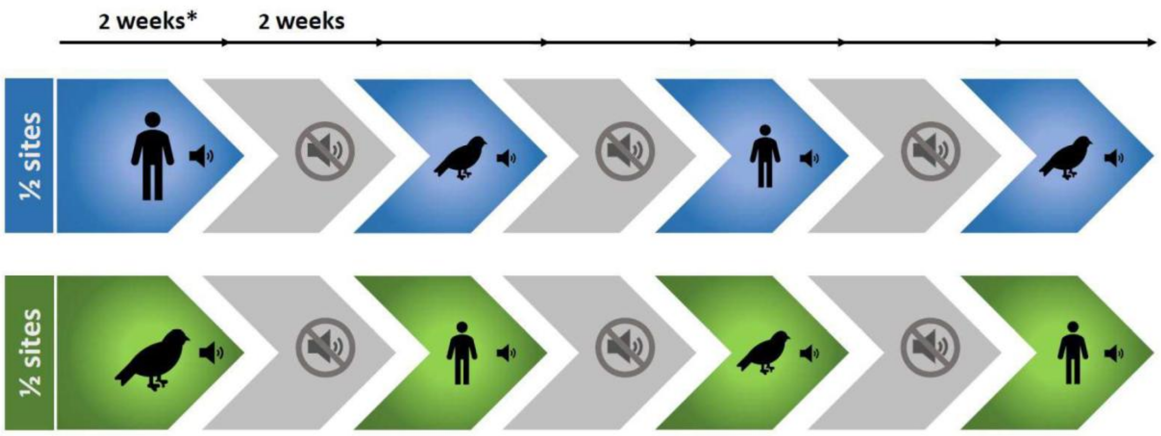

At each test site, wolves were exposed to either the Human voices or Bird vocalizations condition for two weeks (*extended to one month if no wolf passages were recorded), followed by a two-week silent baseline. Conditions were alternated and counterbalanced across sites (48% starting with Human, 52% with Bird). Each condition was presented twice, with two sets of different audio clips (A and B).

Tables

Table S1. Test sites included in the experiment (grouped for location) with year of data collection, Human Footprint Index (HFI), type of test performed (Novel Object or Playback), and the number of individual wolves identified at each location.

Location	Year	HFI	Test site	Test performed	No. individuals
bagnolino	2021	17.770	PCBA	Novel Object	6
catenaia	2021	19.862	CNT16	Novel Object	6
montaccio	2021	24.047	PNOMO	Novel Object	3
sanrossore	2021	23.668	SRIM	Novel Object	2
antinori	2022	30.358	COAB	Playback	1
calvana	2022	26.670	POCC	Novel Object	6
			POCBO	Playback	4
castelvecchio	2022	19.201	VEC1	Novel Object	3
			VECB	Playback	2
gaville	2022	35.099	CEGE	Novel Object	2
germinaia	2022	30.194	PIGBO	Playback	2
migliana	2022	23.899	POM1	Novel Object	5
			POM2	Playback	3
montemuro	2022	28.007	CEML	Playback	3
poggiopapi	2022	36.289	PIPP	Novel Object	2
sanvito	2022	33.174	PISVC	Novel Object	2
			PISVP	Playback	1
torraccia	2022	33.796	COTC	Novel Object	2
			COTP	Playback	1
torreluciana	2022	29.817	COTT	Novel Object	7
vaiano	2022	28.078	POVT	Novel Object	5
casale	2023	13.196	SGCB	Novel Object	10
			SGCS	Playback	1
chiesa di pulicciano	2023	22.724	BSCPB	Novel Object	7
citerna	2023	19.365	BMCM	Novel Object	3
			BMCI	Playback	1
collinaccia	2023	19.792	FCC	Novel Object	7
			FCI	Playback	4
fontefredda	2023	17.784	FFB	Novel Object	3
			FFC	Playback	1
fresciano	2023	24.313	BMF	Novel Object	4
			BMFL	Playback	1
lamartina	2023	20.436	FMA	Novel Object	4
			FME	Playback	4
massicaia	2023	20.526	SGMS	Novel Object	6
			SGMA	Playback	2

monterotondo	2023	21.692	FMC	Novel Object	7
			FMS	Playback	3
poggiotortore	2023	32.226	FPT2	Novel Object	5
			FPTC	Playback	3
sancresci	2023	23.728	BSSCC	Novel Object	5
			BSSCS	Playback	2
serre	2023	32.336	FSB	Novel Object	2
trebbio	2023	27.197	BSCTB	Novel Object	4
			BSCTM	Playback	2
villore	2023	12.766	SGVCU	Playback	3
			SGVCA	Novel Object	3
campiglioni	2024	19.199	DICW	Novel Object	6
			DICH	Playback	2
carbonaia	2024	43.761	ARCR	Playback	1
collamaesta	2024	10.784	MCML	Novel Object	2
			MCMH	Playback	2
crocemori	2024	14.360	DICMH	Novel Object	3
			DICML	Playback	1
lastra	2024	40.398	LSC	Novel Object	2
			LHT	Playback	2
lozzole	2024	14.170	PSLL	Novel Object	3
lucignano	2024	37.310	ARCLU	Novel Object	2
montesenario	2024	25.829	VAMC	Novel Object	3
			VAMH	Playback	2
passoeremo	2024	9.553	MPEG	Novel Object	2
			MPEH	Playback	2
pratoalbero	2024	13.520	PSPAS	Novel Object	1
rata	2024	23.752	DIRH	Novel Object	8
			DIRL	Playback	2
spazzavento	2024	24.876	FISH	Novel Object	2
tirli	2024	14.892	PSTF	Novel Object	2
villetto	2024	12.848	PSVHP	Novel Object	2

Table S2. Ethogram of wolves' behaviors coded.

Behavior	Description
Insecure posture	The back is curved, the head is kept aligned or under the horizontal line of the back, the tail is kept on the vertical line of the legs, lower or even tucked.
Startle	The subject jumps back quickly and suddenly.
Flee	The subject flees (runs away) after looking/interacting with the stimulus (Novel Object test) or after the sound started (Playback test), or after standing or walking (Baseline preceding the Novel Object test).
Wince	The subject draws the body back suddenly, tensing the body, but remaining on the spot.
Slow walk	The subject walks very slowly with cautious movements, stiff back legs, sometimes showing jerky movements or stopping for a few seconds and then walking again.
Risk assessment*	Subject's weight is shifted forward, with muzzle gradually approaching the stimulus and the back legs extended. The subject spreads closer to the object but is ready to jump back in case of danger.
Avoidance*	After having looked at the stimulus or while looking at it, the subject deviates from its straight path, increasing the distance between itself and the stimulus.
Change direction**	The subject changes the direction of movement following the start of the sound.
Stillness**	The subject completely stops any body movement (for at least 2 seconds) following the start of the sound.

Each behavior was coded as presence/absence (1/0). * Coded only for the Novel Object test; **coded only for the Playback test.

Table S3. Full model FearNovObj.

Term	Estimate	SE	*Z or χ2*	*P*	Min	Max
(Intercept)	-1.213	0.300	-4.037	<0.001[e]	-1.465	-1.193
Condition(Object2)[a]	-0.541	0.297	-1.822	0.069[e]	-0.868	-0.622
HFI[b]	-0.442	0.187	-2.361	0.018[e]	-0.443	-0.271
Alone/group(group)[c]	-1.159	0.360	-3.222	0.001	-1.197	-0.892
Age/status(breeding)[d]	-0.163	0.363	-0.449	0.654	-0.232	0.000
Human passage rate[b]	0.010	0.126	0.077	0.939	-0.255	-0.106
Event[b]	-0.926	0.250	-3.705	0.000	-1.027	-0.746
Condition*HFI	0.697	0.286	6.155[f]	0.013[f]	0.606	0.878

[a] Dummy coded with condition Object1 being the reference category; [b] z-transformed to mean=0 and sd=1; [c] Dummy coded with condition alone being the reference category; [d] Dummy coded with age/status as not-breeding being the reference category; [e] these P values have a limited interpretation; [f] the indicated likelihood ratio test refers to the overall effect of the respective interaction (tested by comparing the full model with a corresponding reduced model lacking the interaction).

Table S4. Full model FearHumVoices.

Term	Estimate	SE	*Z or χ2*	*P*	Min	Max
(Intercept)	0.258	0.376	0.685	0.493[d]	-0.035	0.386
Condition(Human)[a]	2.024	0.483	4.186	<0.001[d]	1.949	2.197
HFI[b]	-0.211	0.277	-0.762	0.446[d]	-0.419	-0.026
Alone/group(group)[c]	-1.189	0.450	-2.645	0.008	-1.313	-0.956
Event[b]	-1.046	0.257	-4.067	<0.001	-1.192	-0.914
Condition*HFI	-0.055	0.434	0.016 [e]	0.900 [e]	-0.206	0.158

[a] Dummy coded with condition Bird being the reference category; [b] z-transformed to mean=0 and sd=1; [c] Dummy coded with condition alone being the reference category; [d] these P values have a limited interpretation; [e] the indicated likelihood ratio test refers to the overall effect of the respective interaction (tested by comparing the full model with a corresponding reduced model lacking the interaction). In the main text are reported the values of the reduced model lacking the interaction between condition and HFI.

Table S5. Full model FearNovObj-Voices.

Term	Estimate	SE	*Z or χ2*	*P*	Min	Max
(Intercept)	-1.679	0.303	-5.547	<0.001[d]	-1.777	-1.638
Test(Human)[a]	2.554	0.522	4.892	<0.001[d]	2.414	2.839
HFI[b]	-0.378	0.150	-2.518	0.012	-0.427	-0.317
Alone/group(group)[c]	-0.602	0.315	-1.909	0.056	-0.689	-0.502
Human passage rate[b]	0.046	0.140	0.332	0.740	-0.142	0.075
Event[b]	-1.224	0.332	-3.687	<0.001[d]	-1.370	-1.160
Test*Event	-1.440	0.859	3.092[e]	0.078[e]	-1.939	-1.096

[a] Dummy coded with condition Object1 being the reference category; [b] z-transformed to mean=0 and sd=1; [c] Dummy coded with condition alone being the reference category; [c] Dummy coded with condition alone being the reference category; [d] these P values have a limited interpretation [e]; the indicated likelihood ratio test refers to the overall effect of the respective interaction (tested by comparing the full model with a corresponding reduced model lacking the interaction). In the main text are reported the values of the reduced model lacking the interaction between test and event.

Movie S1 (separate file). Example videos of wolves tested in the Novel Object test and Playback test. https://figshare.com/s/a439bb50a8e50f1fb84b?file=55758653

Dataset S1 (separate file). Raw data used for the analyses.

Dataset S2. (separate file). R scripts.
https://figshare.com/s/a60d6fcbf09b0c882dd5

Dataset S3. (separate file). R workspace.
https://figshare.com/s/a60d6fcbf09b0c882dd5

SI References

1. H. Mu, X. Li, Y. Wen, J. Huang, P. Du, W. Su, et al. A global record of annual terrestrial Human Footprint dataset from 2000 to 2018. *Sci Data* **9,** 176 (2022).
2. C.E. Jacobs, D.E. Ausband. An evaluation of camera trap performance – What are we missing and does deployment height matter? Remote Sens Ecol Conserv. **4**, 352–60 (2018).
3. L. Mattioli, A. Canu, D. Passilongo, M. Scandura, M. Apollonio. Estimation of pack density in grey wolf (Canis lupus) by applying spatially explicit capture-recapture models to camera trap data supported by genetic monitoring. Front Zool. **15**, 38 (2018).
4. B.M. Clarin, E. Bitzilekis, B.M. Siemers, H.R. Goerlitz. Personal messages reduce vandalism and theft of unattended scientific equipment. *Methods Ecol Evol.* **5**:125–31 (2014).
5. M.S. Palmer, C. Wang, J. Plucinski, R.M. Pringle. BoomBox: An Automated Behavioural Response (ABR) camera trap module for wildlife playback experiments. Methods Ecol Evol. **13**, 611–8 (2022).
6. D. Wheat. Arduino Software. https://doi.org/10.1007/978-1-4302-3883-6_5
7. Vegas Pro. Vegas Pro. Sony Creative Software; 2020. https://www.vegascreativesoftware.com
8. O. Friard, M. Gamba. BORIS: a free, versatile open-source event-logging software for video/audio coding and live observations. Methods Ecol Evol. **7**:1325–30 (2016).
9. C.L. Larson, S.E. Reed, A.M. Merenlender, K.R. Crooks. Effects of Recreation on Animals Revealed as Widespread through a Global Systematic Review. *PLOS ONE*, **11**:e0167259 (2016).
10. Core Team R. A language and environment for statistical computing. R Found Stat Comput (2021)
11. D. Bates, B.M. Bolker, S.C. Walker. Fitting linear mixed-effects models using lme4. *Journal of Statistical Software* **67**: 1-48 (2015)
12. H. Schielzeth. Simple means to improve the interpretability of regression coefficients. Methods Ecol Evol. **1** :103–13 (2010).
13. J. Fox, S. Weisberg. An R companion to applied regression (Sage publications, 2011).
14. G.P. Quinn, M.J. Keough. Experimental design and data analysis for biologists (Cambridge university press, 2002).
15. D.J. Barr, R. Levy, C. Scheepers, H.J. Tily. Random effects structure for confirmatory hypothesis testing: Keep it maximal. *J Mem Lang.* **68**, 255–78 (2013).
16. H. Schielzeth, W. Forstmeier. Conclusions beyond support: overconfident estimates in mixed models. *Behav Ecol.***20**:416–20 (2009).
17. W. Forstmeier, H. Schielzeth. Cryptic multiple hypotheses testing in linear models: overestimated effect sizes and the winner's curse. *Behav Ecol Sociobiol.***65**, 47–55 (2011).
18. A.J. Dobson, A.G. Barnett. An introduction to generalized linear models (CRC press, 2018).
